# Formation of Cystine Slipknots in Dimeric Proteins

**DOI:** 10.1371/journal.pone.0057443

**Published:** 2013-03-08

**Authors:** Mateusz Sikora, Marek Cieplak

**Affiliations:** Institute of Physics, Polish Academy of Sciences, Warsaw, Poland; Weizmann Institute of Science, Israel

## Abstract

We consider mechanical stability of dimeric and monomeric proteins with the cystine knot motif. A structure based dynamical model is used to demonstrate that all dimeric and some monomeric proteins of this kind should have considerable resistance to stretching that is significantly larger than that of titin. The mechanisms of the large mechanostability are elucidated. In most cases, it originates from the induced formation of one or two cystine slipknots. Since there are four termini in a dimer, there are several ways of selecting two of them to pull by. We show that in the cystine knot systems, there is strong anisotropy in mechanostability and force patterns related to the selection. We show that the thermodynamic stability of the dimers is enhanced compared to the constituting monomers whereas machanostability is either lower or higher.

## Introduction

The cystine knot motif is an interlaced structural arrangement involving three cystins, i.e. three pairs of cysteines connected by disulfide bonds. Two of these cystins effectively transform two short segments of the backbone into a closed ring. The third one connects two different parts of the backbone through the ring [1],[2]. This tight structure provides remarkable thermodynamic stability. It has been first observed in a nerve growth factor (NGF) [3] and then identified in other growth factors [5]. It has also been found in small cysteine-rich toxins [2], where it was found to stabilise the structure of small cyclic peptides to a greater extent than the cyclisation of the backbone [5]. These toxins remain stable and active at temperatures nearing the boiling point or at large concentrations of chemical denaturants and enzymes.

The cystine knot motif is highly conserved and is a part of many growth factors [6], [7]. The growth factors are involved in the development, tissue differentiation and healing processes in all phyla. In mammals, for instance, there are over 30 different kinds of TGF-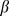 proteins which control these processes. In particular these proteins can be potential drug targets in cancer therapy [8], [9].

Growth factors are usually flat and extended. They lack a hydrophobic core and expose the hydrophobic residues to the solvent. The growth factors typically form dimers [10]. Upon dimerisation, the exposed hydrophobic residues become burried and generate attractive contact interactions which bind the monomers in conjunction with up to two intermeric disulfide bonds. The dimeric structures are thus rigid and stable in the solvent. Additionally, residues involved in the cystine knot form an evolutionaly conserved framework of amino acids responsible for interactions with receptors of the growth factors [10]. For instance, in the transforming growth factors-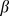 (TGF-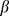) family the receptor binding sites reside near the cystine knot motif whereas the remaining residues are quite variable and lead to the phenomenon known as receptor promiscuity [8], [11]. This phenomenon results in the ability to bind to distinct receptors, while still maintaining the capacity to dimerize the receptors and initiate the TGF-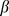 signalling pathway [12].

It should be noted that growth factors are also involved in mechanical processes. It has been shown [8], [13], that the growth factors are secreted by a cell in the form of pre-proteins with a hydrophobically attached long peptide which intertwines with the growth factor. The peptide enhances solubility. In the process of maturation, the peptide appears to detach by mechanical forces exerted by the extracellular matrix. The released growth factor interacts with corresponding receptors and via proteins of the SMAD-family releases transcription factors, influencing activity of the cell.

The mechanical stability of proteins with cystine knots has not been assessed experimentally yet. However, theoretical studies of proteins with cystine knot, based on coarse grained [14] and all-atom models [15] indicate that it may be significantly higher than that of titin or ubiquitin. Specifically, the characteristic force, 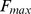, needed to unravel the tertiary structure may be in the range of even 1 nN, i.e. about five times as big as for titin [16], and yet smaller than a force needed to break a covalent bond [17], . For the sake of comparison, 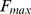 of between 800 and 900 pN has been reported for the protein molecule in the spider capture-silk thread [19].

The parameter 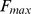 is determined by stretching a protein at a constant speed and observing the largest force peak when plotting the force, *F*, against the pulling spring displacement, *d*. For most proteins, the force peaks are due to shear between two or more 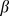-strands [20]–[22]. On the other hand, for the proteins with cystine knots, 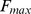 arises due to formation of a cystine slipknot that takes place when the ring-piercing disulfide bond drags a segment of the backbone through the ring. It should be noted that the cystine slipknot is distinct from the protein slipknot such as studied in protein AFV3-109 [23]. The cystine slipknot is formed dynamically whereas the slipknot in AFV3-109 is present in the native state and pulling gets it untied in multiple pathways. We have found that the top 13 strongest proteins, among the 17 134 simulated [14], [22], are endowed with the cystine slipknot mechanism. There also a hundred of such proteins with a smaller mechanostability. The protocol used in the theoretical studies involved taking into consideration the first structure which is associated with a given Protein Data Bank (PDB) code [24] – either the first chain or the first NMR-based model. It has turned out, however, that many of the cystine slipknot proteins are dimers and that this feature has much bigger dynamical consequences than in the case of non-cystinic complexes. These circumstances call for reinvestigation of the behavior of proteins with the cystine knot during stretching. In this paper, we show that such dimeric proteins are indeed remarkably stable mechanically, various variants of the slipknot mechanisms are operational, and that the response to stretching, even the very existence of force peaks, strongly depends on the choice of the two out of four terminal amino acids to pull by (non-terminal points of force attachment add to the variety of choices). Such a highly anisotropic dependence on the selection of the termini has been predicted for the titin Z1Z2-telethonin complex [25], [26] and, more recently [27], for the 3*D* domain-swapped cystatin [28] – the dimeric protein without any cystine knots. In both of these cases, however, the mechanical clamps involved are common as they are due to shear.

### Geometry of the Systems Studied

Here, we focus on four main families of proteins that contain growth factor cystine knots (GFCKs) – one of the three known kinds of cystine knots (the other being inhibitor cystine knot and cyclic cystine knots) [1], [2], [29]. These are TGF-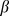, NGF, glycoprotein hormones (GPH), and platelet-derived growth factor (PDGF). The latter family has a branch of vascular endothelial growth factors (VEGF; its human homologues are known as PlGF – placenta growth factors). Table 1 lists the specific structures we investigate here, together with their family affiliations and the values of 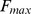 for various ways of pulling. 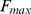 is in units of 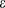/Å, which should be of order 110 pN, where 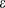 is the depth of the potential well associated with each native contact. Throughout the paper, we use the theoretical units to allow for an easier comparison with our surveys. Table 1 contains 8 proteins belonging to the TGF family and 6– to the VEGF family. These proteins coincide with the 13 top strength structures found in the mechanostability survey of monomers [14]. In addition, we consider one protein, 1TFG, which should have been at the top part of the list as well if not for its mistaken removal from consideration by structure filtering algorithms. (We have excluded from considerations the PDGF BB protein with the structure code 1PDG, as this structure is incomplete). Table 1 lists two structures associated with the PDB code 1M4U: chain L and chain A and hence the corresponding subscripts. 1M4U_A_ is known as noggin whereas 1M4U_L_ studied in ref. [14] is a ligand of noggin and is known as BMP-7 protein (bone morphogenetic protein). _L_ belongs to the TGF family and its properties are similar to those of 1BMP. Noggin, on the other hand, is a signalling protein involved in many developmental processes such as neural induction and bone development [36]. It acts through inactivation of the ligands belonging to the TGF-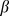 family. Each of the chains L and A separately forms dimers and each monomer contains a cystine ring. The ring in 1M4U_A_ is wider: it contains 2 more residues than in 1M4U_L_. If 1M4U_A_ and 1M4U_L_ bind then a tetramer forms.

**Table 1:** Values of 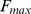 of the proteins studied here in units of 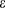/Å and for different pulling schemes.

PDBid	Family	Sr		FM	FN−C′	FN−N′	FC−C′	FN−C	ΔTf
dimeric									
1BMP	TGF	8		10.3	3.4	−	4.0	−	0.02
1LXI	TGF	8		7.3	4.0	−	3.5	−	0.02
2BHK	TGF	8		7.3	3.5	−	3.7	−	0.01
2GH0	TGF	8		5.9	8.6	−	12.0	−	0.03
2GYZ	TGF	8		5.4	4.6	−	5.5	−	0.03
1REW	TGF	8		5.3	4.5	−	3.2	−	0.02
1M4UL	TGF	8		5.3	8.0	−	8.0	−	0.01
1TFG	TGF		8	5.5	14.2	1.1	14.0	1.1	0.02
1QTY	VEGF		8	8.9	5.6	4.6	−	1.9	0.04
1CZ8	VEGF		8	6.4	5.7	4.9	−	5.8	0.04
1FLT	VEGF		8	5.5	5.2	4.4	−	4.7	0.03
1WQ9	VEGF		8	5.5	4.5	4.7	−	6.1	0.03
1FZV	VEGF		8	5.4	4.5	4.2	−	5.5	0.03
1VPF	VEGF		8	5.3	5.9	5.0	−	5.8	0.04
1M4UA	noggin		10	2.8	2.5	2.6	−	1.5	0.02
1BET	NGF		14	2.1	1.6	2.4	2.1	3.4	0.02
1HRP	HCG		8	−	3.4	2.0	3.0	−	0.02
monomeric									
lefty	TGF		8	4.1					
1IXT	knottin		8	2.2					
1W7Z	knottin		11	1.2					
1H20	knottin		9	2.2					
1JU8	knottin		8	5.8					

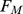
 denotes 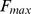 in the monomeric case – when only one chain of the dimer is considered in the N-C mode. The four penultimate columns are for the the dimeric situation. The subscripts of 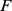 indicate the mode of pulling. 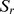 denotes the number of amino acids in the cystine ring. The last column shows a difference between melting temperature of a dimer and two separated monomers, 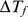 in units of 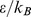.

**Figure 1:**
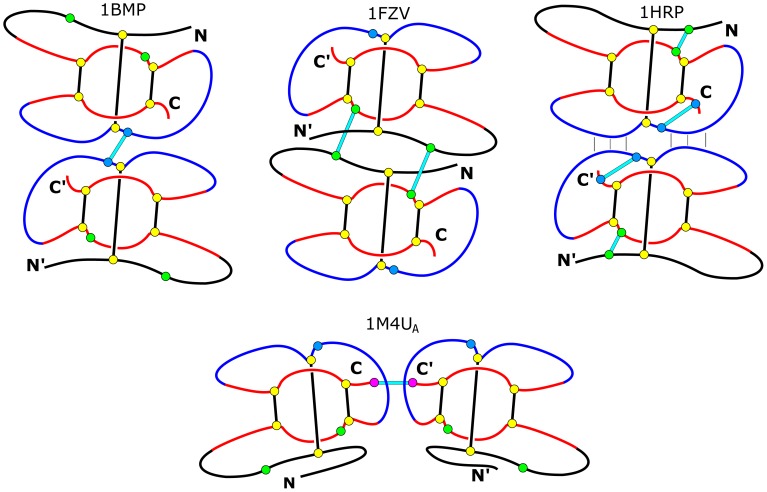
Schematic representations of four types of dimer architectures as exemplified by structures 1BMP and 1FZV, representing the TGF and VEGF families respectively and structures 1M4U_A_ and 1HRP as indicated. The cysteines involved in the cystine knot motif are shown as yellow circles. Other relevant sites are shown as circles either in blue, green, or magenta. In at least one of the four families shown here, these sites are occupied by cysteines – this happens if the circles are connected by lines, i.e. disulfide bonds. For instance, in 1BMP the green circles do not show cysteines but the sites that would house cysteines in the structure corresponding to 1FZV. The symbols N,C and N′, C′ in the drawings do not indicate locations of the terminal amino acids since the corresponding backbones do not end at these places. Rather, they indicate amino acids which are sequentialy closest to the indicated termini. The intra-monomer disulfide bridges are represented by thick black lines, whereas the inter-monomer bridges are shown as lines in cyan. The monomers in 1BMP and 1M4U_A_ are connected through one cystine but in two different ways. In 1M4U_A_ the cystine effectively links the rings as it provides connection of Cys230 on the ring through the nearby C termini Cys232 to Cys230′ on the other ring. In 1BMP it links amino acids just next to the ring-piercing cysteins. In 1FZV there are two binding cystines. Each of them links a ring in one monomer with an N-proximal segment in the other monomer. For 1BMP and 1FZV the rings comprise 8 amino acids. In the case of 1M4U_A_–10 amino acids. In the case of 1HRP, The vertical lines between two monomers indicate hydrophobic contacts and hydrogen bonds.

Figure 1 shows schematic connectivities of proteins belonging to the TGF and VEGF families, as represented by 1BMP and 1FZV structures respectively. It also shows the scheme of connectivities in 1M4U_A_. In the latter case, the two monomers are linked through the cysteinic termini C and C′ which are one residue away from a cysteine on the ring. In the case of 1BMP, the chain-connecting cystine (with Cys103) is just next to the cysteine (Cys104) that forms a ring-piercing disulfide bond with a cysteine (Cys38) which is sequentially near the N-terminus (the known structure extends between sites 36 and 139). The 1FZV dimer has two cystins that bridge the monomers. Each of them links a ring in one monomer with an N-proximal segment belonging to the other monomer.

**Figure 2:**
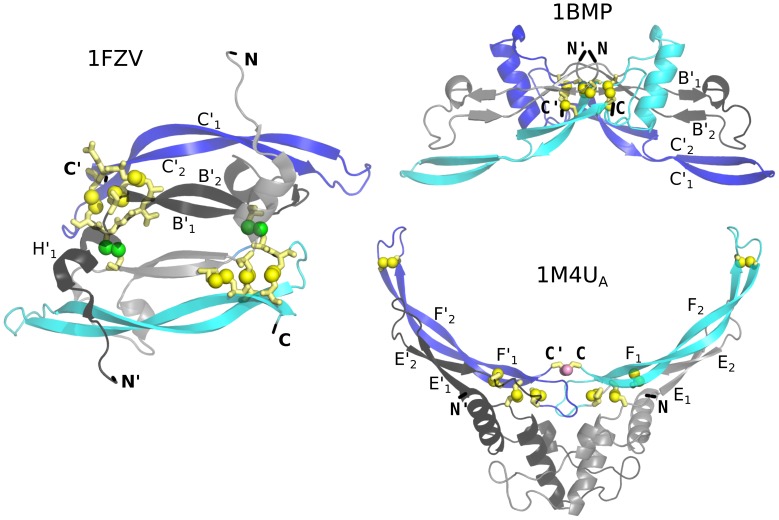
The molecular representation of the native dimeric structures 1FZV, BMP, and 1M4U_A_ as shown by the labels. The termini are indicated. The unprimed symbols refer to one monomer and the primed symbols to the other. The terminal amino acids are indicated in black. The yellow spheres correspond to the atoms of sulfur belonging to the cystine rings. In the panel corresponding to 1FZV, the green spheres correspond to the cysteines that link the two monomers. In the panel corresponding to 1BMP, the atoms of sulfur in the cystines that link the two monomers are hidden behind the yellow spheres. In the panel corresponding to 1M4U_A_, the inter-monomeric disulfide bond is indicated by two spheres in pink; one of them is in front of the other.

More realistic representations of these three native dimeric structures are shown in figure 2. The overal elongated geometry of a GFCK monomer is well conserved in the examples shown and is perhaps best seen in top left panel corresponding to 1FZV. The structure is dominated by a long, 2-stranded 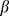-sheet (strands 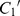 and 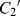) that span the whole length of the protein. It is accompanied by a shorter 2-stranded sheet (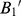 and 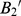). In the case of 1BMP, both sheets are partitioned into two segments as seen in the top right panel. The cystine knot is located close to one of the ends of the monomer and seems to act as as separator between the sheets. While the geometry of a monomer changes a little between the specific proteins, we observe that the dimeric proteins employ three schemes to connect. In the case of the VEGF proteins, the monomers are arranged antiparallelly, as shown for 1FZV in the top middle panel of figure 0, which allows for formation of two connecting disulfide bonds. In the case of the TGF proteins, the monomers are connected at an angle and then only by one disulfide bond as illustrated for 1BMP in the top left panel. Finally, the monomers may be just touching in a small region where they form a disulfide bond that connects the C and C′ termini. This situation happens in 1M4U_A_, as illustrated in the bottom panel of figure 2.

The inter-meric connectivities discussed so far are provided primarily by disulfide bonds but additional linkages can arise due to hydrophobicity and hydrogen bonds forming between the monomers. An alternative strategy to bind is not to involve the cystines. This situation happens for 1HRP and the corresponding connectivities are indicated in the top right panel of figure 1. A similar pattern is also valid for 1BET.

For completness, we have also studied five monomeric proteins with the cystine knots. They are listed as the bottom entries in Table 1. One of them, denoted as lefty, is a member of the nodal family of signaling factors. It is expressed during embryo development and is responsible for formation of the left-right axis [37]. The monomeric character of lefty is not certain but it is likely to be valid [38]. The remaining entries are exceptionally stable short peptides known as knottins, all sharing a cystine knot motif, but interlaced differently than in the families of growth factors [29]. This motif still contains a cystine that pierces a ring. A knottin is formed by the cystine knot that is stripped off any surrounding secondary structures. Here, we do not study a subgroup of knottins in which the backbone is circular which makes the proteins ultra stable.

### The Modeling Procedure

The modeling is done within the coarse grained dynamical model used in ref. [14], described in more details in refs. [30]–[32].The starting point is to form a polymeric chain of beads that are tethered together by a harmonic potential. Each bead represents an amino acid. The disulfide bonds are covalent and are also represented by the harmonic potentials. We account for the local backbone stiffness by introducing 4-body terms which favor the native sense of the local chirality, which is nearly equivalent to favoring native values of the dihedral angles. The remaining interactions are defined in terms of contacts: native and non-native. The native contacts are determined based on the overlap of the van der Waals spheres assigned to heavy atoms and the 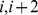 contacts are discarded as they are usually weak. Our structure-based modeling relies on assigning pair-wise binding potentials to two amino acids that are linked by a native contact and assigning repulsive potentials to all other pairs of amino acids, i.e. to the non-native contacts. We consider a soft repulsive potential which acts when the distance, 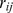 between the C^α^ atoms is less than 4 Å. The condition on the binding potentials is that their minima should correspond to the experimentally determined native distance between the C^α^ atoms in the contact-making amino acids. There are countless ways in which such potentials can be constructed. However, only some of them lead to unobstructed folding to the native state and to consistency with the experiments on stretching. We have considered 62 models and tested them against the experimental values of 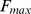 obtained for 38 systems [32]. We have identified four models which are optimal. Here, we use the simplest of the four in which the binding potential has the Lennard-Jones form
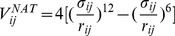
(1)in which the energy parameter 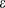 does not depend on the identity of the amino acids whereas the length parameters, 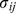, are selected so that the minima of the potentials agree with the native distances. The callibration of  is obtained by finding the best fit to the experimental data on 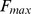 and is given by [14] ∼110 pN Å that has been mentioned in the previous section. The predictions of this model have been positively verified by stretching experiments on two scaffoldin proteins [33].

The molecular dynamics simulations of stretching are done at temperature 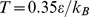, which should correspond to a vicinity of the room temperature (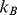 is the Boltzmann constant) and is in the region of good folding. Stretching is accomplished by attaching a spring at each of the two selected termini considered. The other end of one spring is anchored and that of another is made to move with a constant speed. The pulling speed is of order 0.005 Å/ns. This speed is within the range of some stretching experiments [14], but is some two orders of magnitude faster than typical stretching exepriments at slower speeds. Nevertheless it is some five orders of magnitude slower than typical all-atom simulations. In our regime of speeds, 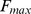 depends on the speed merely logarithmically, so estimates at 0.005 Å/ns are meaningful. The extrapolation to experimentally accessible pulling speeds yields 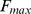 by about 10% smaller. For instance, we estimate that for 2GH0 the force of 12.0 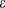/Å (∼1320 pN) gets reduced to to 10.45 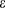/Å (∼1150 pN) when lowering the speed from the theoretical 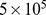 nm/s to the expected experimental speed of 500 nm/s. The spring constant is taken to be 0.12 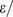
Å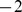 which is of the order of typical AFM lever elastic constants. We have found [30] that the choice of the spring constant influences the way the 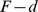 pattern is spread but it has only a minor effect on the value of 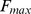. When studying extraction of bacteriorhodopsin from a membrane [34], an agreement with the look of the 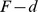 pattern was obtained by reducing the theoretical spring constant by a factor of 1.35. For each case studied, we have considered up to ten trajectories and most typical behavior was chosen for a display in the figures. The value of 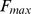 is averaged.

In addition to the mechanical stability, we also assess thermal stability. The thermal stability of a protein can be characterized by the folding temperature, 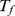. It can be defined computationally [35] in a long equilibrium run as a temperature at which the probability, 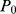, of staying in the native state crosses 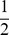. One may argue that the system can be considered as staying in the native state when all of its native contacts are present. However, it is a matter of choice to declare at which distance between a pair of the C*^α^* atoms a contact is still operational. Our criterion is to take the inflection point in the contact potential as providing a working threshold.

## Results and Discussion

### Assessment of Mechanostability

For each of the proteins listed in Table 1, the termini extend out to the solvent and could thus be grasped in experiments involving single molecule manipulation [39], [40] easily. If N and C refer to the termini of the first dimer partner, and N′ and C′ to the second then there are four choices to select pairs of termini in which one terminus is anchored and another is pulled. These choices are indicated by the symbols N-C′, C-C′, N-N′, and N-C (due to the symmetric arrangement of cystine rings in both monomers, N′-C′ yields same results as N-C, etc.). In this notation, N-C′ means anchoring the N terminus of the first monomer and pulling by the C-terminus of the second monomer. Anchoring at C′ and pulling at N yields the same result. The dependence of 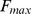 on the choice of attachment points has been discussed for monomeric proteins, for instance, in refs. [41], [42].

**Figure 3:**
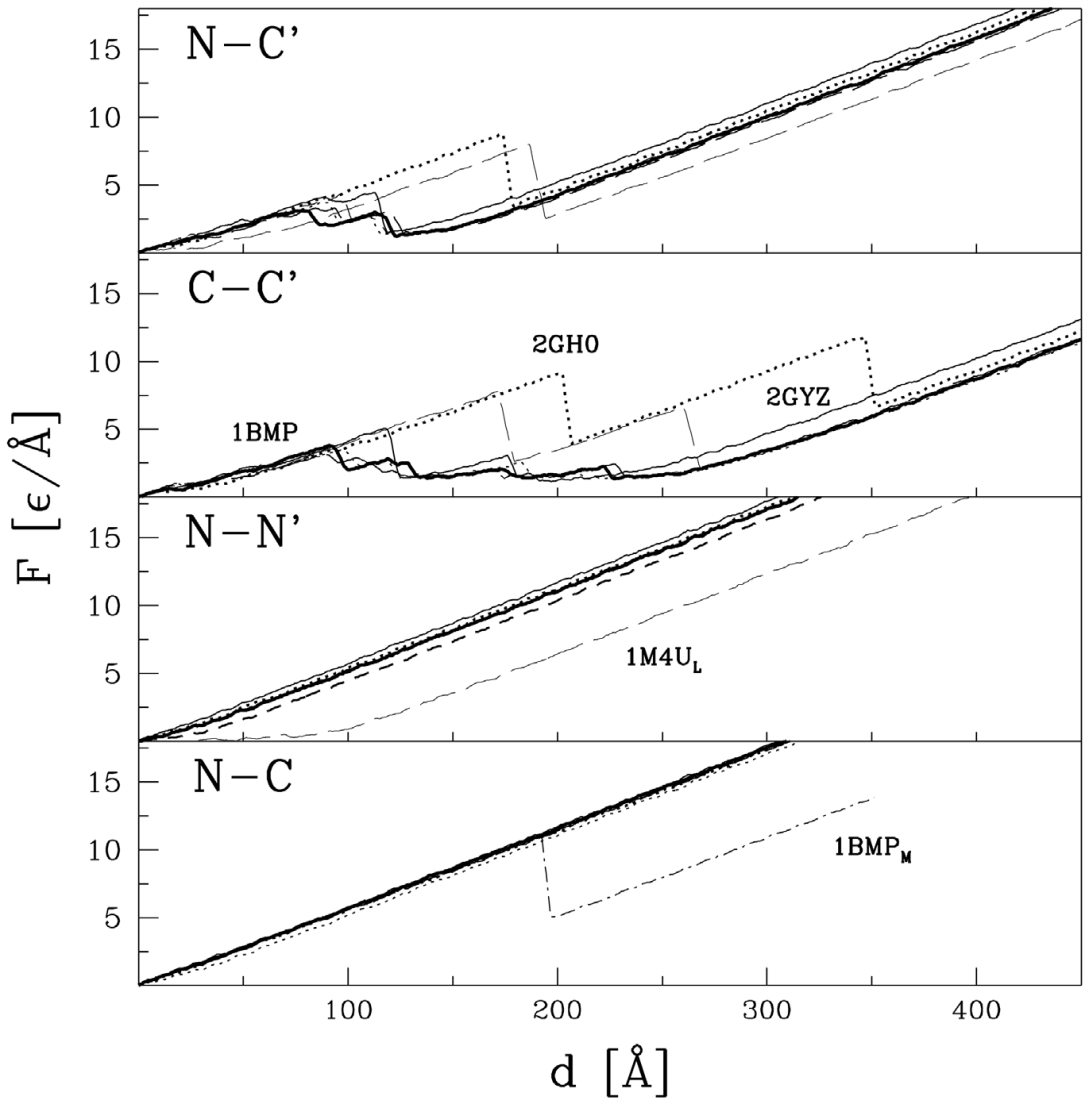
The 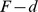 curves for the proteins of the TGF family that are listed in Table 1. The ways of pulling are indicated in the upper left corner of each panel. The line with the symbol 1BMP_M_ in the lowest panel indicates the result for a single monomer, if extracted from the dimer. For other curves, the line type for a given protein is the same in each panel.

**Figure 4:**
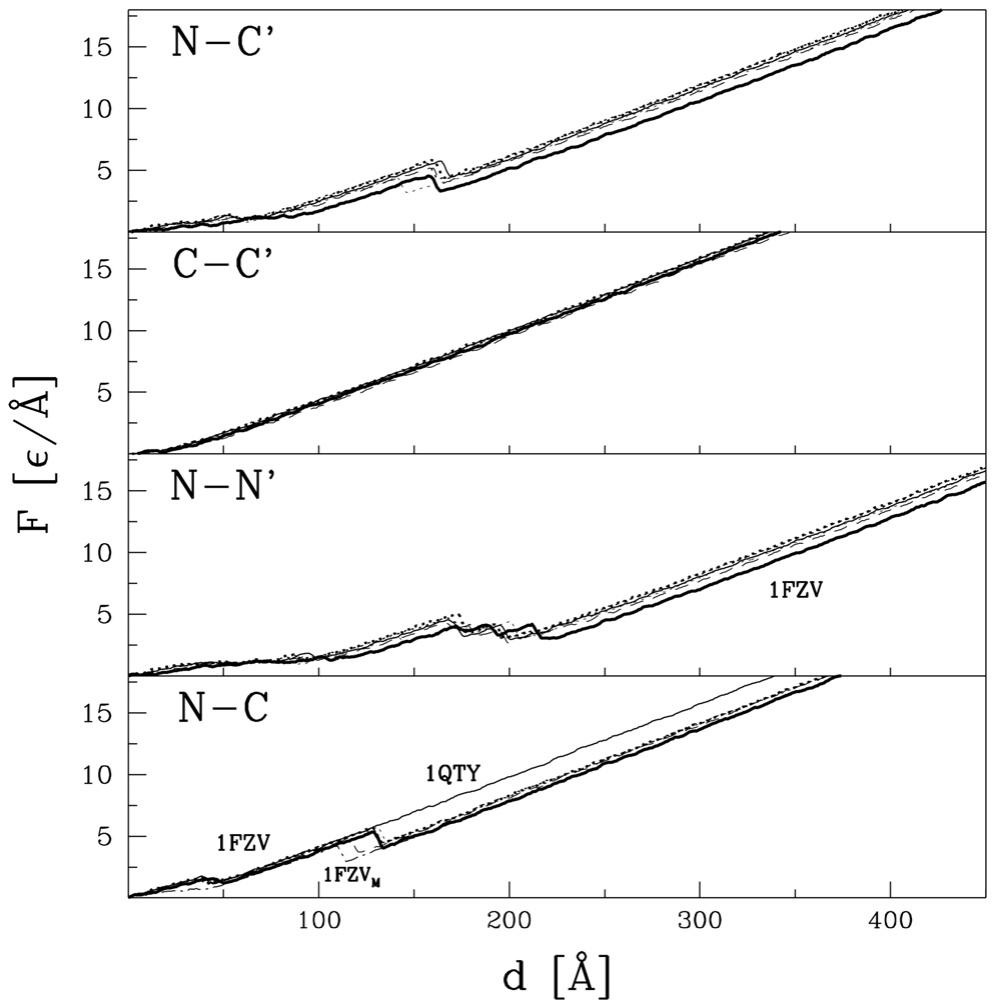
Similar to figure 2 but for the VEGF proteins listed in Table 1.

Figures 3 and 4 show the 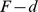 curves for the TGF and VEGF proteins, that are listed in Table 1, respectively. We observe that for each way of pulling, the curves within the TGF family are similar to each other and so are the curves within the VEGF family. In particular, there are no force peaks in the TGF proteins when pulled within the N-N′ and N-C schemes and no force peaks in the VEGF proteins when pulled in the C-C′ way. Whenever force peaks do arise, their heights, shown in Table 1, are similar within families and across families. They are about 5 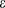/Å, i.e. of order 550 pN. The exception is 2GH0 for which 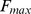 is twice as big. It should be noted that the 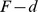 curves appear to have essentially no curvature for large values of 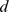, past the last force peak, whereas a finite curvature is predicted by the worm-like-chain model [43]. The reason is that the theoretical model applies to entropic chains in which potential energy contributions are negligible. This is not the situation encountered at such high tensions as considered here though some curvature might become perceptible when going to considerably larger extensions.

**Figure 5:**
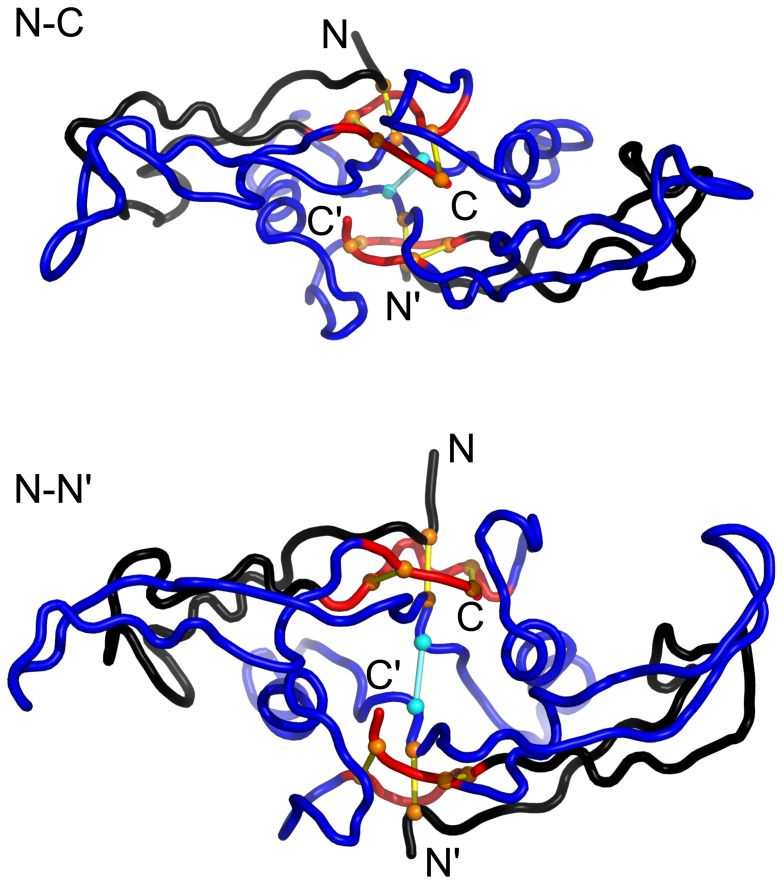
Examples of stretched conformations of 1BMP for the NC (top panel) and NN′ (bottom panel) pulling at *d* = 250 Å. peaks arise through manipulations of these kinds. The color coding is similar to that used in Figure 1. In the top panel, the N terminus pulls on the blue loop of the first monomer. The resulting force is transferred to the second monomer through the inter-molecular cystine bridge that is shown in cyan. The second monomer is too big to cross the cystine ring and, therefore, the tension grows indefinitely, exceeding values needed to break covalent bonds. In the bottom panel, stretching results in an immediate allignment of the three cystine bridges: within the cystine knots (in yellow) and the intermolecular one, and in an indefinite growth of the tension.

**Figure 6:**
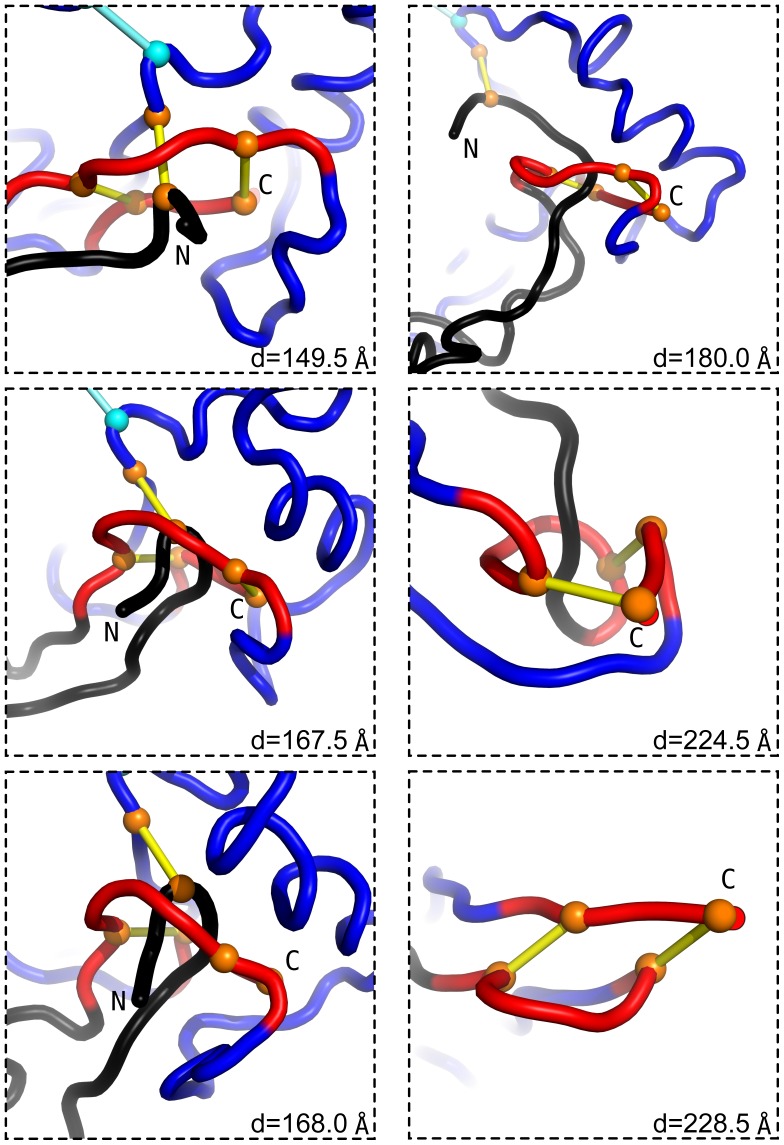
Subsequent snapshots of the model 1BMP during the C-C′ stretching. The corresponding values of *d* are indicated. The figure shows only the region in which the cystine slipknot forms. The first of the frames shows the knot near it’s native state. In the middle left panel the knot loop (shown in black) approaches the inside of the ring. In the next snapshot, it squeezes halfway through the ring. This is the stage corresponding to the highest tension reached during unfolding. In the next frame (top right), the loop has already slipped past the ring. At this point, the system is unable to return to the native state rapidly if the pulling spring is removed. Two subsequent frames show further extension of the protein. In the bottom right panel, the whole length of the slip-loop has crossed the ring: the slipknot is released. The second force peak is due to the formation of the cystine slipknot in the second monomer (not shown).

Insights into the nature of the pulling process can be obtained by monitoring transformations in the conformations. This is illustrated for 1BMP in Figures 5 and 6 as well in a movie available in the Supplementary Information. Figure 5 addresses situations in which isolated force peaks do not arise (N-C and N-N′ pullings) and figure 6 is for the C-C′ pulling when they do. Figure 6 shows six subsequent stages of the process.

**Figure 7:**
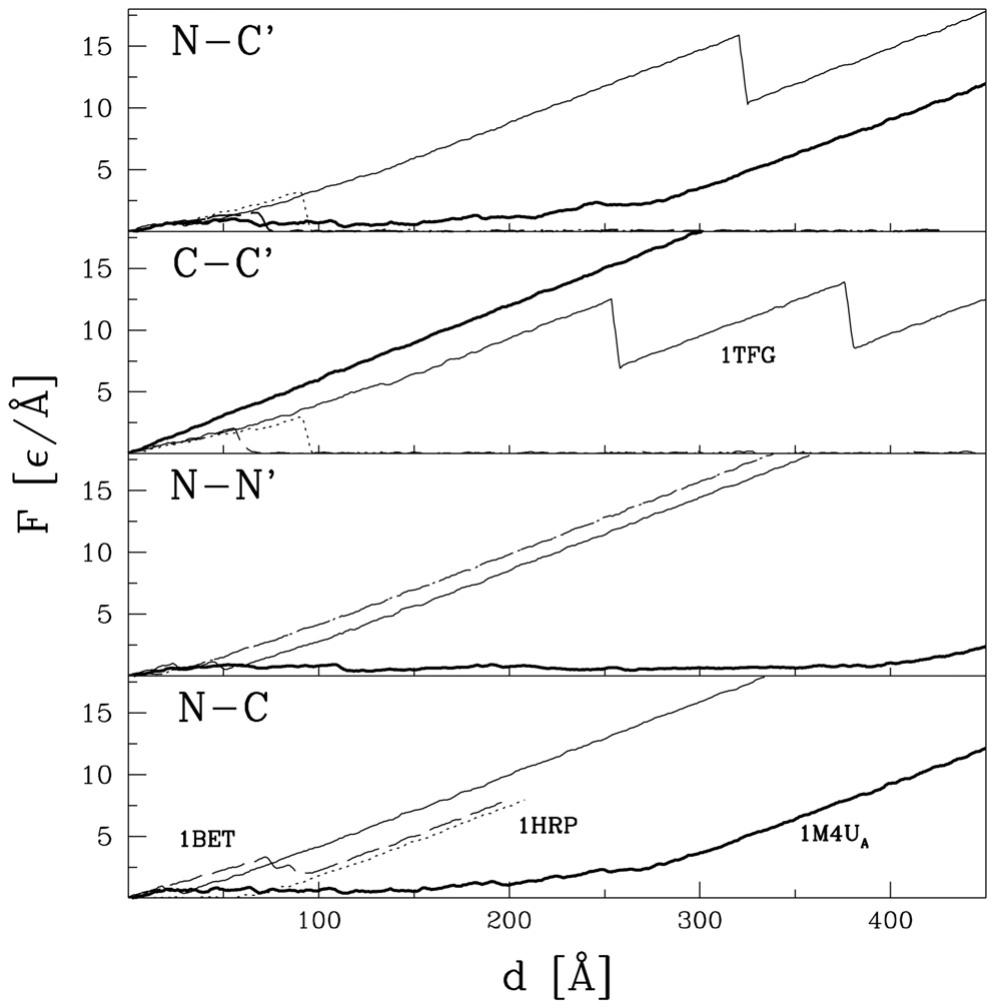
Similar to figures 3 and 4 but for 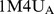 and the remaining proteins with cystine knots listed in Table 1.

The 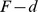 plots for noggin, shown in figure 7, are similar to those for the VEGF family (figure 4) in the sense that no force peak develops in the C-C′ scheme, but the force peaks appearing within other schemes of pulling are minor.

We now consider proteins, 1BET and 1HRP, in which the bridges between monomers are not cystinic. For the N-C pulling, 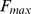 is equal to 3.4 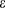/Å in the case of 1BET, but there is no force peak for 1HRP. For other kinds of pulling there is an eventual separation of the monomers which is preceded by development of a force peak resulting from overcoming the shear. Even though the cystine ring in 1BET consists of more residues than in 1HRP, the largest force peaks for both are similar in height: 3.4 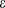/Å. These force peaks are related to the shear between the monomers and they do not involve formation of any slipknot.

**Figure 8:**
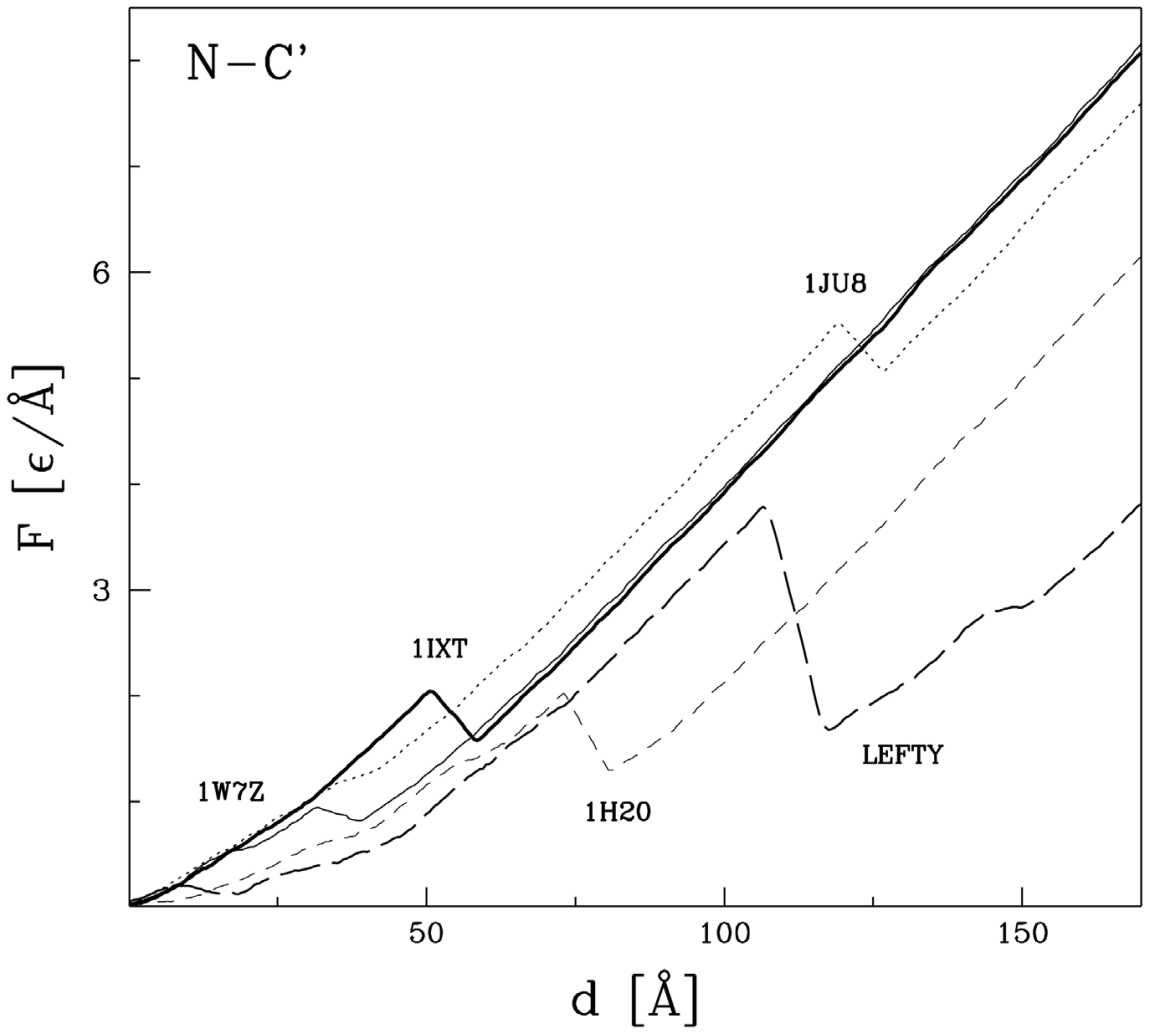
The 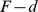 curves for the monomeric proteins with a cystine knot that are listed in Table 1. The stretching is accomplished by the termini.

The 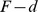 curves for the monomeric knottins are shown in figure 8. Each such curve comes with a single peak which involves dragging of a slipknot through the ring. The largest mechanostability in this set is displayed by 1JU8 (a 4-kDa peptide found in legumes). The corresponding 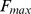 is about 5.8 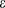/Å. This is a remarkably large force, considering that 1JU8 consists of only 37 residues (the survey in ref. [14] has dealt with proteins of at least 40 residues).

**Figure 9:**
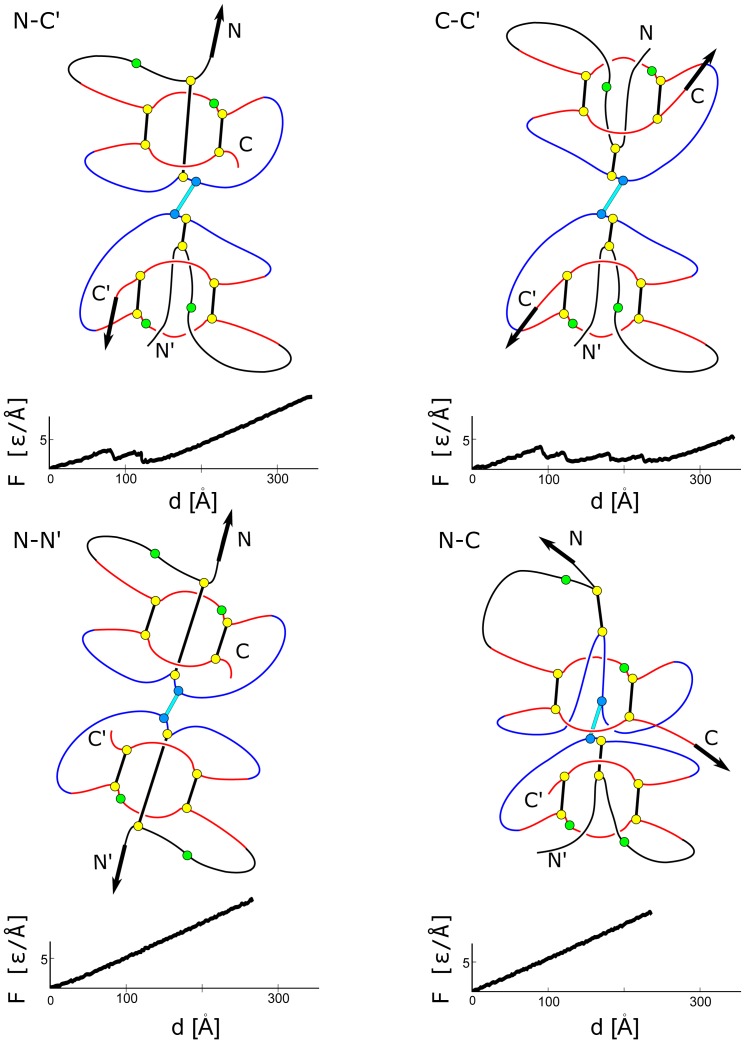
Mechanisms involved in stretching of a protein from the TGF family for the four choices of attachment points. Below each panel, there is a corresponding 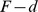 plot obtained for 1BMP.

**Figure 10:**
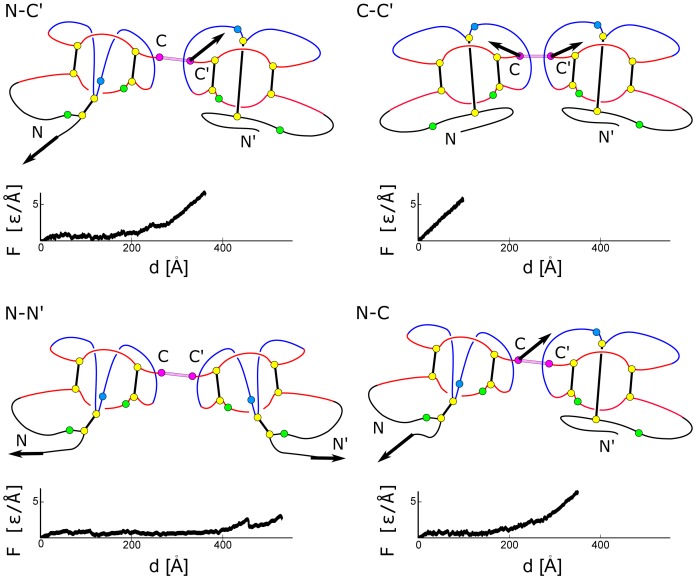
Mechanisms involved in stretching of noggin (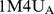) for the four choices of attachment points. Below each panel, there is a corresponding 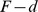 plot.

**Figure 11:**
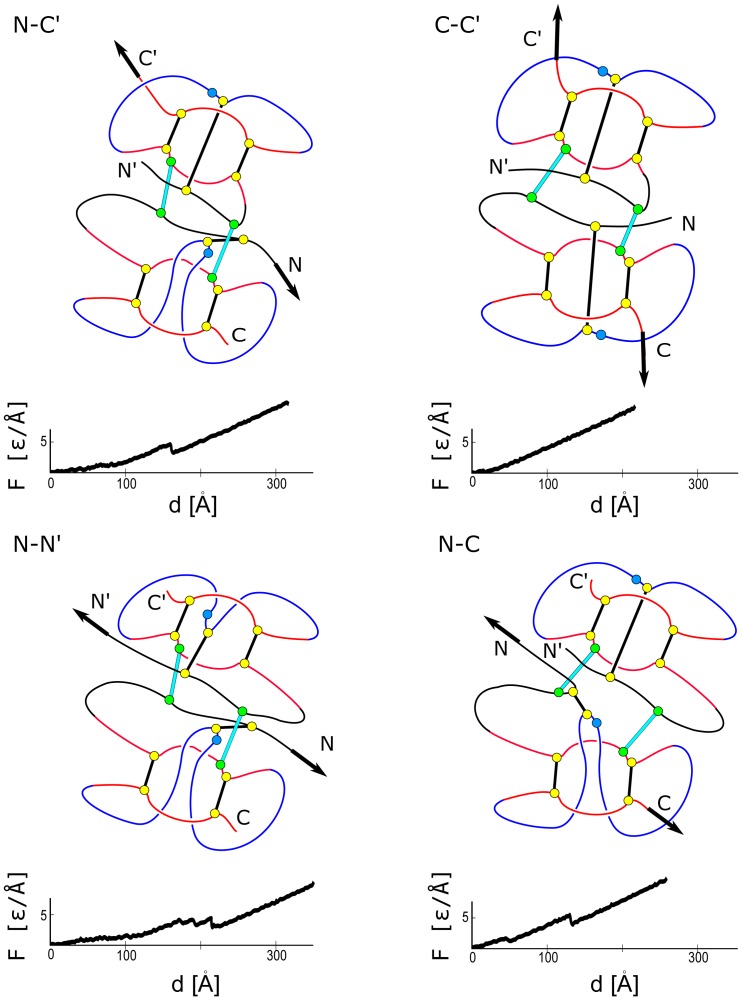
Similar to figures 9 and 10 but for a protein from the VEGF family. The 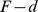 plots are for 1FZV.


The mechanisms involved in stretching are illustrated in figures 9, 10, and 11 for 1BMP (representing TGF), 1M4U_A_, and 1FZV (representing VEGF) respectively. The figures also show the corresponding 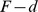 plots. We first consider stretching of 1BMP. There are no force peaks for the N-N′ and N-C ways of pulling as the tension builds up, indefinitely, only in the covalent bonds (along the backbone and in the disulfide bonds) – see also figure 5. In the case of the N-N′ stretching, the system stiffens immediately on pulling as the ring piercing cystines and the monomer connecting cystine align in series (the lower left panel of figure 9) without rupturing any native contacts and without formation of a slipknot – only the covalent bonds get stretched. In the case of N-C, the situation is more subtle. The slipknot in the first monomer cystine ring does form but it does not go all the way through to generate a force peak before initiating a process of tension relaxation after overcoming a bottleneck. The reason is that dragging of the slipknot is halted by the second monomer which would have to go through the ring, but it is too big to squeeze in. Pulling in the remaining cases, N-C′ and C-C′, generates articulated force peaks because the pulling direction is not parallel to the cysteine that connects the monomers. This results in non-simultaneous passages of two slipknots. In the case of C-C′ (see also figure 6, the force peaks are split further because of shearing in a 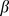-sheet near the N-termini. After the pivotal part of the knot loop squeezes through the ring, it is held for an instant by the hydrogen bonds between strands 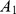 and 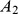 shown in figure 2 These contacts need to be broken to negotiate threading of the knot loop through the ring. The same scenario is observed in the 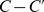 pulling. However, it is repeated twice as both monomers act symmetrically.

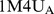
 is structurally similar to the TGF proteins but the monomers are linked at the C and C′ termini which results in no force peaks for the C-C′ way of pulling. For the remaining modes of stretching, one or two (in the case of N-N′) slipknots form but the resulting peaks are barely observable (see figure 10). This is because the cystine ring consist of 10 residues instead of 8 and thus provides less hindrance to the motion of a slipknot.

The mechanisms of stretching in the VEGF proteins are outlined in figure 11. Notice that the dimer has a two-fold symmetry with respect to the axis perpendicular to the plane containing all four termini (thermal fluctuations make this symmetry approximate). Thus pulling within schemes N-N′ and C-C′ results in a motion which is symmetrical with respect to the axis and in equal tensing of the two inter-monomer bridges. In the case of N-N′ this means nearly simultaneous generation of two slipknots whereas in the case of C-C′ no force peaks. In the other two cases, there is no symmetry and a single slipknot is generated leading to a noticeable force peak.

### Comparison to Single Chain Stretching

**Figure 12:**
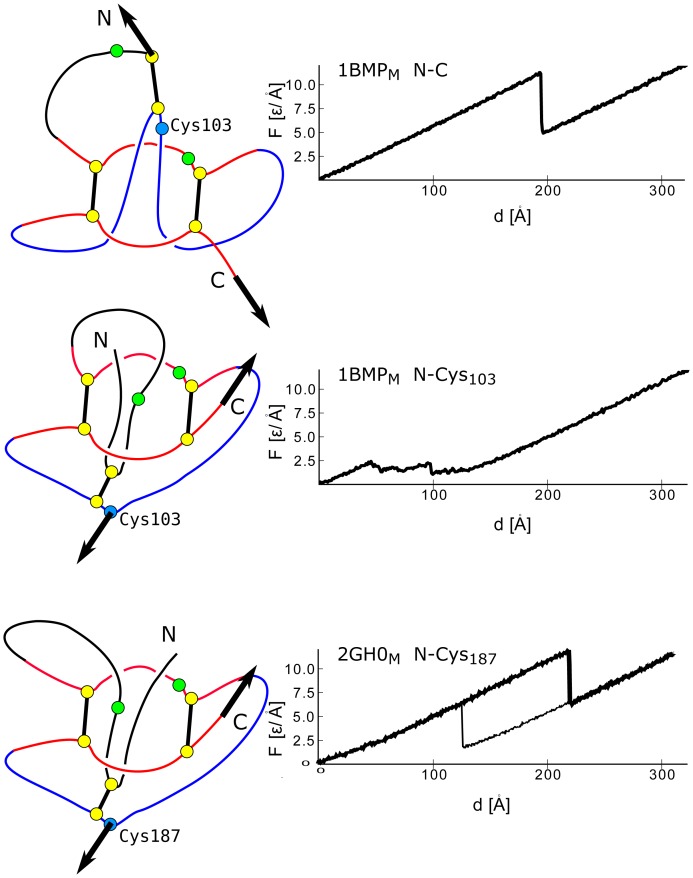
Top two rows of panels: Comparison of pulling of the monomeric 1BMP at different points of attachment of the pulling force. Bottom row of panels: stretching of the monomeric 2GH0. The thick force line is for the N-Cys187 pulling and the thin force line is for a similar situation, in which, however, the contacts between the knot-loop (strands 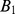 and 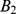 in figure 2) and the rest of the protein is removed. These contacts affect the angle at which the ring piercing cystine is dragged across the ring: they make the pulling at a small angle between the plane of the ring and the plane of the slipknot. In the absence of these contacts, the approach is more vertical which results in the observed reduction of the force.

**Figure 13:**
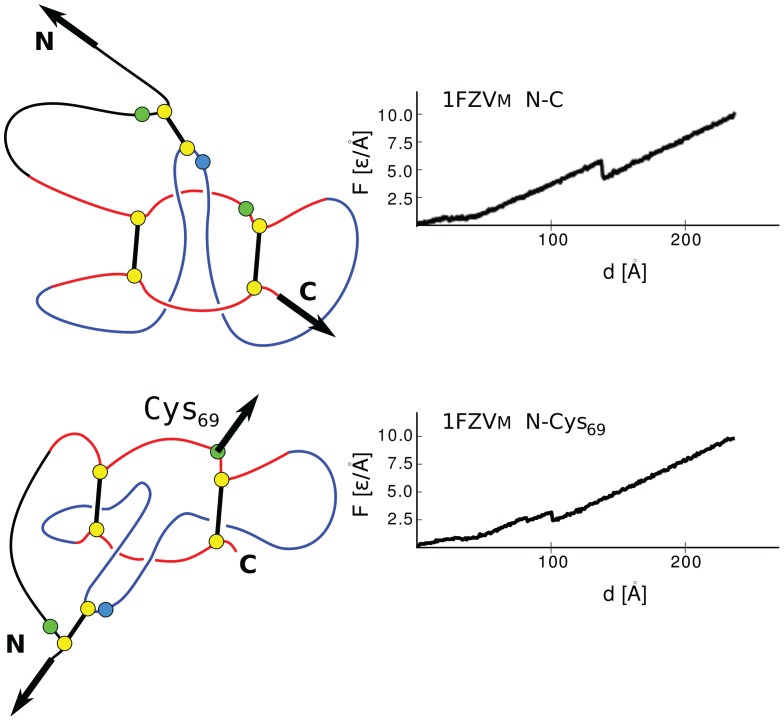
Stretching of the monomer of 1FZV at different points of force attachment. The top panel shows the conventional stretching of a monomer by N and C termini. This kind of manipulation is inaccessible when the protein is in its active, dimeric state. Stretching in the dimeric N-C case, however, results in effectively shifting the location of the pulling force from the C-proximal cysteine on the ring to another cystine, Cys69, on this ring. The deformation of the ring is different and, in addition, the slip-loop crosses the ring more vertically. Both of these factors result in an overall reduction in the force in the dimeric N-C stretching.

Table 1 shows the values of 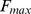 for the dimeric situations but also compares them to those obtained assuming that a monomer is not connected to any partner. For some proteins (2GH0, 1M4U_L_, 1TFG, 1WQ9, 1VPF) the monomeric values of 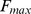 are smaller than the largest 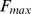 for the peak-bearing dimeric cases. For some (2GYZ, 1FZV) – almost the same. In the remaining cases we observe lowering of 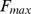 – even by a factor of 2 as for 1BMP. The short explanation for the observed differences is that even though monomeric and dimeric peak forces are due to cystine slipknots, dragging of the knot-loop usually takes place in different directions (e.g. opposite) or different angles with respect to the cystine ring. These circumstances can be explained with the help of figures 12 and 13 for 1BMP and 1FZV respectively. The top panels refer to the monomeric pulling in the N-C way which produces dragging of the slipknot towards the N terminus which is sequentially close to one partner of the cystine that does the dragging.

In the dimeric 1BMP, the force peaks arise in the N-C′ and C-C′ stretchings which results in dragging of the slipknot by Cys103 in the opposite direction and in pulling of the N-terminus into the ring. The effect on the 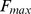 is a four-fold reduction in the value when this kind of manipulation is induced in the monomeric 1BMP. The reduction is also related to a more vertical dragging of the slipknot through the ring which results in a smaller deformation of the ring. The double peak is due to letting the N-terminus that is followed by sliding of the knot-loop through. The situation is quite similar for other TGF proteins. A bigger difference occurs for 2GH0, where Cys187 acts as the pulling agent. This difference is that the N-terminus does not enter the ring on the rim side. Instead, the structure of the protein is such that the slipknot is rotated so that the N-terminus enters near the center, requiring more space to pass without separating the passage into the distinct knot-loop and N-terminus events. In this case, 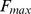 gets doubled. The physics of the slipknot formation in 1TFG is quite special as it involves dragging of a ring through a ring which we dubbed the mechanism of the cystine plug. We deal with it with in a separate publication [44].

For the N-C′ pulling of the dimeric 1FZV, the relevant forces are effectively attached to N and Cys69 on the cystine ring. (Cys69 transports most of the tension between the monomers.) The resulting slipknot still protrudes towards the N-terminus, as in the N-C pulling of the monomer, but it makes a different angle with respect to the ring (the bottom panels of 12) which results in a reduction of 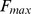.

We now come back to figure 11 and observe that when the dimeric 1FZV is stretched in the symmetric N-N′ way, there are, surprisingly, three consecutive force peaks. Judging by the symmetry, we would expect to find an even number of peaks. We could explain this puzzle by a selective switching of certain contacts off and investigating the effect of such an action. Each monomer of this protein encompases two characteristic 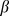 sheets, each containing two strands, as shown in Fig. 2 In 1FZV, the longer sheet (strands 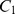 and 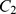) spans the whole length of the protein comprising 17 amino acid on each of the strands. When the protein is dimerised, the sheet is facing the outer side of the complex. The shorter of the 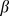 sheets (*B*_1_, *B*_2_) runs parallel to the long one and contains 8–10 amino-acids. In the dimeric state, it faces the inter-monomer interface but it does not form a larger sheet with its symmetric counterpart. The two sheets are bridged together by a cystine ring. We have found, that shearing of the bonds within the shorter stretch of 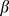 sheet is responsible for the emergence of the first and smaller peak. This process arises in both monomers simultaneously. Indeed, the first of the peaks disappears upon removal of contacts between the two shorter 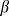 sheets in each monomer (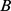 and 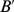). Clearly, these contacts are not only responsible for the first peak, but they also contribute to the remaining two peaks, as their removal reduces the height of the remaining two peaks. To summarise, in the monomeric 1FZV, 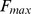 is almost entirely due to steric hindrance but in the dimeric 1FZV there is also a contribution from attractive native contacts.

### Monomeric Cystine Knot Proteins

We now consider the cystine knot proteins that are monomeric. One example is knottins. Figure 8 shows the 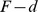 curves for four examples of such proteins. The first example is a toxin extracted from from a sea snail *Conus gloriamaris* [45]. This protein has the structure code 1IXT and it comprises only 27 amino-acids. Structurally, it is related to the family of cyclic disulfide-rich peptides. Despite its short length, our simulations of stretching suggest existence of an articulated force peak with 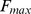 of order 2.2 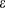/Å which just exceeds 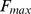 of titin when calculated within the same model [14]. The second example is the tripsin inhibitor protein II (code 1W7Z) derived from the poisonous squirting cucumber *Ecballium elaterium*. which shares the cystine-knot topology. In contrast, it yields only a minor force peak with 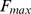 of 1.2 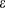/Å. The smaller force is due to the larger size of the cystine ring – it consists of 11 residues instead of 8 as in 1IXT. The 39-residue potato carboxypeptidase with the code 1H20 is found to be as strong as 1IXT. The biggest 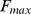 in the set considered is predicted to arise in the 31-residue long leginsulin (code 1JU8). The corresponding 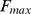 of about 5.8 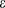/Å is as big as for the most robust dimeric cystine knot proteins listed in Table 1.

Another example of monomeric cystein knot systems is a group of proteins from the nodal signaling pathway which governs cell differentiation in embryonal development [37] such as lefty, for which an existence of monomers was suggested. No structure of lefty protein has been solved to date. We have thus resorted to a homology model, that has been calculated using ModPipe software [46] using a standard set of parameters. It should be noted that the disulfide bridge between cysteins 251 and 253 in the derived structure was above a distance treshold and had to be set manually. We predict that 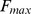 for lefty (see figure 8) should be nearly twice as big as for conotoxin: 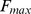 = 4.1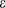/Å. In all examples considered in this section, the force peaks arise due to formation of the cystine slipknot conformation. The hypothesis that growth factors form dimers due to the lack of the hydrophobic core appears not to be valid in the case of the monomeric lefty, which is the member of the same family. However, no structure of this protein has been solved so the judgement should be suspended.

### Non-mechanical Stability

We now come back to the dimeric cystine knot proteins. One may compare the largest value of 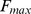 obtained in one of the four ways of stretching the dimers to 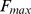 derived for stretching of an extracted monomer. By looking at the values listed in Table 1, we realize that the difference between the two forces, 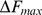, can be either positive or negative. In contrast, as evidenced by the last column, the thermodynamic stability resulting from joining two monomers into a dimer is always enhanced. The change in thermal stability, 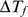, is defined as the difference in 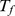 between the dimer and two separate monomers. The degree of this enhancement is not related to 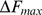. For instance, the largest 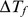 of 0.04 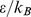 (about 35 K) is for 1QTY and 1VPF whereas 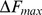 for these two proteins is 3.3 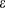/Å and merely 0.6 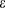/Å respectively. The values of 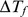 were inferred based on five long trajectories. Our results are related to the outcome of experiments on the VEGF proteins carried out by Muller et al. [47]. The authors have mutated particular cysteines within the cystine knot and found out that this action results in worse folding and somewhat reduced thermal stability. Here, on the other hand, we assess the effect of dimerization on thermal stability of the GFCK proteins and conclude that the inter-monomer disulfide bridges have similar effect, i.e. they increase the thermal stability. Furthermore, a two-bridge connectivity (as in the TGF proteins) tends to provides a bigger thermal stability than a one-bridge connectivity (as in the VEGF proteins). Another way to see the role of the cystins is by reducing all of the disulfides. This has been accomplished for artemin (2GH0) [48]. The resulting lowering of the folding temperature was of order 40 K. Yet another study investigates the role of inter-monomer disulfide bridges [49], [50] in PDGF. It has been found on mutating the cysteines in the intermeric disulfide bridges into serines does not prevent the dimer from forming. However, its resistance to chemical denaturation and changes in pH is reduced dramatically.

The TGF-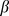 proteins are known to display very slow chemical denaturation [51], [52]. It may last for hours and refolding is even slower. The corresponding rates increase linearly with temperature. Interestingly, the authors of ref. [51] have proposed an unfolding mechanism which is essentially similar to the cystine slipknot clamp but defined for chemical/thermal unfolding. In their mechanism, the slip-loop is slowly dragged out of the cystine ring as a result of entropic effects. In our model of mechanical stretching, the time scales are several orders of magnitude too short to observe such fluctuational processes. On the other hand, the slow nature of the refolding processes seen experimentally explains the irreversibility of unfolding observed in our studies.

### Concluding Remarks

In this paper, we have shown that the primary mechanical clamp associated with the force peaks in proteins with the cystine knots is formation of the cystine slipknot, independent of whether the proteins are monomeric or dimeric. In our calculations, we include no attractive non-native contacts in the model. Whereas they might play a role in folding, their effect can only be minor in the context of the cystine slipknot mechanism which is dominated by steric interactions. We have elucidated the workings of the cystine slipknot mechanical clamps in dimeric systems and demonstrated emergence of interesting topological transformations. We have shown that dimeric systems with the cystine knot should be giants of mechanostability like the corresponding monomeric systems, but the picture is more subtle since generating large force peaks requires stretching only in certain directions. Furthermore, the action of this kind of clamp in dimers may be different from that in monomers. For instance, dragging of a single slip-loop may take place in different direction than in the corresponding monomer and the resulting 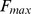 may be either smaller or larger than in the monomer.

It is probably unlikely that forces of this magnitude affect proteins with the cystine knots under biological conditions. However, our studies may motivate research in bio-inspired materials containing such proteins as building blocks. These materials would behave similar to the spider dragline as they could absorb and dissipate large ammounts of energy. Simple polymers, even if very stiff, do not develop force peaks on the 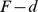 curves whereas the energy which is absorbed and then dissipated depends on the area under the peaks. Crossing a peak locks the system (practically irreversibly) in a stretched conformation and generates an element akin to a sacrificial bond. In this way, fibers or networks made of such proteins should be able to withstand forces larger than those associated with systems made of simple polymers.

Exploration of such bio-inspired materials should be preceded by an experimental verification of our findings on mechanostability.

## Supporting Information

Movie S1
**The first movie shows unfolding of the 1BMP dimer in the C-C′ mode.** One monomer is colored in blue and the other in red. The cystine bonds are indicated as yellow sticks. The arrows indicate amino acids through which the pulling process is implemented. The whole duration of the movie corresponds to 100 1000 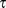, where 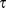 is of order 1 ns.(AVI)

Movie S2 **The second movie is a close-up that is focused on the workings of the cystine slipknot clamp in action.** The coloring of the backbone segments is the same as used in the main text. Halfway through, an attempted but unsuccessful passage of the slip-loop can be seen. The attempt becomes successful only after the tension crosses the threshold value. The movie ends at about half of the total duration of the first movie, when all slip-loop has crossed the ring and no further structural rearrangement is possible.
(AVI)
